# The beneficial effects of leave extract of *Commiphora leptophloeos* on intestinal mucositis induced by 5-fluorouracil in mice

**DOI:** 10.1590/acb411526

**Published:** 2026-04-10

**Authors:** Amanda Silveira da Silva, Stephannie Jamyla de Araújo Barbosa, Susana Barbosa Ribeiro, Lucas Vinicius Souza de Carvalho, Rafaela Torres Dantas da Silva, Gerlane Coelho Bernardo Guerra, Raimundo Fernandes de Araújo, Nizyara Costa da Silva, Raphael Victor Silva Andrade, Gabrielle Marques de Paiva, Silvana Maria Zucolotto Langassner, Renato Dantas-Medeiros, Rafaela Alcindo Silva de Sousa Fé, Maria Laura de Souza Lima, Aurigena Antunes de Araújo

**Affiliations:** 1 Universidade Federal do Rio Grande do Norte – Department of Pharmacy – Natal (RN), Brazil.; 2 Universidade Federal do Rio Grande do Norte – Department of Biophysical and Pharmacology – Postgraduate Program in Pharmaceutical Science – Natal (RN), Brazil.; 3 Universidade Federal do Rio Grande do Norte – Department of Biophysical and Pharmacology – Postgraduate Program in Biotechnology – Natal (RN), Brazil.; 4 Universidade Federal do Rio Grande do Norte – Departament of Morphology – Postgraduate Program in Health Science – Natal (RN), Brazil.; 5 Universidade Federal do Rio Grande do Norte – Department of Morphology – Postgraduate Program in Functional and Structural Biology – Natal (RN), Brazil.; 6 Universidade Federal do Rio Grande do Norte – Department of Dentistry – Postgraduate Program in Oral Science – Natal (RN), Brazil.; 7 Universidade Federal do Rio Grande do Norte – Department of Biophysical and Pharmacology – Postgraduate Program in Oral Sciences – Natal (RN), Brazil.

**Keywords:** Drug Therapy, Fluorouracil, Mucositis

## Abstract

**Purpose:**

This study aimed to evaluate the effect of oral administration of a hydroethanolic extract of *Commiphora leptophloeos* leaves (CLF) on the progression of 5-fluorouracil (5-FU)-induced intestinal mucositis.

**Methods:**

Female Swiss mice were orally administered CLF at doses of 10, 100, or 300 mg/kg, or saline (negative control, NC), starting 30 minutes before intraperitoneal injection of 5-FU. A positive control (PC) group received 5-FU without CLF treatment. Treatment continued until euthanasia, three days after 5-FU injection. Body weight was monitored throughout the experiment. On day 4, small intestine tissue samples were collected for histopathological analysis. Duodenal tissue was also assessed for Muc-2 immunostaining.

**Results:**

In the CLF 10, 100 and 300 mg/kg groups, the duodenum histopathological score was 1 (1–1.5) *p* < 0.001, 1 (1–1.5) *p* < 0.01; 1 (1–1.25) *p* < 0.01, while the PC group score was 2 (2–2.75). Immunostaining analysis for Muc-2 revealed a score of 3 (3–3) in the CLF 10 group, compared to score 1.2 (0.8–1.6) in the PC group (*p* < 0.001), indicating that CLF 10 increased Muc-2 expression.

**Conclusion:**

The oral administration of a hydroethanolic extract of CLF protected the duodenal segment of Swiss mice from 5-FU-induced intestinal mucositis, likely by increasing mucin production.

## Introduction

5-Fluorouracil (5-FU) is a widely used chemotherapeutic agent in the treatment of various cancers, particularly colorectal cancer. Its antitumor effect results from the inhibition of essential biosynthetic processes or incorporation into macromolecules such as DNA and RNA, thereby interfering with their normal function^1^. However, one of the major adverse effects associated with 5-FU is intestinal mucositis, characterized by symptoms such as diarrhea, nausea, abdominal pain, and gastrointestinal ulceration^2^.

Intestinal mucositis is marked by significant epithelial damage, activation of the nuclear factor NF-κB, and the release of pro-inflammatory cytokines, including tumor necrosis factor (TNF)-α, interleukin (IL)-1β, and IL-6. In addition, this condition results in profound histopathological changes, such as crypt loss, villous atrophy, impaired epithelial renewal, and disruption of intestinal absorptive and barrier functions^3^.

In this context, *Commiphora leptophloeos*, popularly known in Brazil as “imburana-de-espinho,” is a plant native to the Caatinga biome with broad geographic distribution, especially in northeastern Brazil. In traditional medicine, its leaves, fruits, latex, and flowers are used for therapeutic purposes in treating renal disorders, colds, cough, bronchitis, dysphonia, general inflammation, oral conditions, colic, and diarrhea^4^. It is also used as an antiemetic, tonic, and wound healer^5^.

Considering the reported pharmacological properties of *C. leptophloeos*, this study aimed to investigate the potential protective effects of its leaf extract against 5-FU-induced intestinal mucositis in a murine experimental model.

## Methods

### Plant material

*C. leptophloeos* leaves (CLF) were harvested in April 2023 in the Ferreiro community in São Fernando, Rio Grande do Norte, Brazil. A voucher specimen (RN8248) was deposited at the Herbarium of Parque das Dunas, located in Natal, Rio Grande do Norte, Brazil. The collection was carried out under authorization from the Brazilian Biodiversity Authorization and Information System (SISBIO – permit no. 35017) and registered with the Genetic Heritage Management Council (Environment Ministry) under SISGEN code A618873.

### Preparation of hydroethanolic extract

The leaves were dried in a forced-air oven at temperatures not exceeding 45°C and then ground using a knife mill. The powdered material was subjected to maceration using a 70% hydroethanolic solution in a 1:10 (g/mL) ratio. The maceration process was conducted in two 48-hour cycles. The resulting mixture was filtered through Whatman^TM^ No. 1 filter paper. Subsequently, the solvent was removed under reduced pressure using a rotary evaporator at 35°C. The remaining aqueous extract was then freeze-dried at 200 mT and -60°C for 48 hours. The final product was stored at -20°C until use.

### Pretreatment of sample

The CLF were treated using solid phase extraction (SPE; Chromabond, 45 μm, 500 mg, 6 mL). The cartridges were pre-activated with MeOH (3 mL) and equilibrated with H_2_O (3 mL). An amount of 50 mg of the dry extract was dissolved in EtOH/H2O (85:15 v/v), loaded to a C-18 cartridge, and eluted sequentially with 3 mL of the same mobile phase. The solvent was dried under N2 atmosphere

### Animals and sample size

Female Swiss mice (*Mus musculus*), weighing 25–30 g (mean age of 8 weeks) were housed in polypropylene boxes and kept under controlled temperature (24 ± 2°C) and relative air humidity conditions (50 ± 5%), 12-h light/dark cycle and *ad libitum* access to food and water. Animals were obtained from the animal facilities of the Biosciences Center of the Universidade Federal do Rio Grande do Norte, in Natal. All experimental protocols were approved by the Ethics Committee on the Use of Animals of the Universidade Federal do Rio Grande do Norte (Protocol No. 040/2022) and performed in accordance with the ARRIVE ethical guidelines. All methods were performed in accordance with relevant guidelines and regulations.

The number of animals per group was determined according to the sample size Eq. 1:


(1) n=DF/k+1

It can be used for three common analysis of variance (ANOVA) designs applicable to animal studies, where: k: number of groups; n: number of subjects per group; DF: degrees of freedom^6^.

In view of the ethical considerations that recommend the sample size refinement in studies with animals, we chose to use the minimum number of animals (five animals/group) to carry out the experiments.

### Experimental intestinal mucositis

Intestinal mucositis was induced as previously described^7^. Briefly, a single dose of 450 mg/kg of 5-FU (Libbs Pharmaceuticals LTD., São Paulo, SP, Brazil) was intraperitoneally administered, and animals were euthanized 12 days later by overdose of ketamin (240 mg/kg) and xylazin (30 mg/kg).

### Experimental and control groups

To investigate the impact of CLF on 5-FU-induced intestinal mucositis, three groups of five animals received once-daily oral administration of CLF [doses: 10 mg/kg (CLF10) or 100 mg/kg (CLF100) or 300 mg/kg (CLF300)], starting 30 minutes before the 5-FU administration, until euthanasia, three days after the 5-FU injection. The control animals were divided into two control subgroups (n = 5/group): a group of healthy animals, not submitted to 5-FU-induced intestinal mucositis, that received once-daily administration of saline solution until euthanasia and a single intraperitoneal injection of saline solution on day 1 (saline group-CN); and a group of animals submitted to 5-FU-induced intestinal mucositis that received once-daily administration of saline solution from day 0 to day 3 (5-FU group/positive control-CP). All animals were euthanized four days after the 5-FU injection.

The animals were monitored daily up to the fourth day for signs of moribundity and mortality, such as lack of responsiveness to manual stimulation, immobility, and/or an inability to eat or drink.

### Body weight, peripheral blood leucocyte counts, and hematological analysis

Body weight and peripheral blood leucocyte counts were assessed to identify potential systemic toxicity associated with 5-FU. Body weight was measured on days 1 and 4 of the experiment. Before euthanasia, blood samples (20 μL) were collected from the heart puncture of anesthetized animals. These samples were then diluted in 380 μL of Turk’s solution. Manual counting of total leukocytes was performed using a Neubauer chamber, with the results presented as the number of white blood cells per mm^3^ of blood. Aspartate aminotransferase (AST)/transaminase glutamic-oxaloacetic (TGO), alanine aminotransferase (ALT)/transaminase glutamic-pyruvic (TGP), urea and creatinine levels were determined in serum using standard enzymatic colorimetric methods, following the manufacturer’s instructions, and expressed in U/L (for transaminases) and mg/dL (for urea and creatinine).

### Histopathological analysis

Following euthanasia, tissue samples (including the mucosal, submucosal, muscle, and serosa layer) of each small intestine sections (duodenum, jejunum, and ileum) were collected and fixed in 10% neutral-buffered formalin, dehydrated, and embedded in paraffin for immunohistochemistry and histopathological analysis. Sections (5-µm thick) were obtained for hematoxylin and eosin staining (H&E) and for subsequent light microscopy examination (200x). The severity of mucositis was evaluated in a single-blinded fashion and graded using a modification of the MacPherson and Pfeiffer’s histopathological grading system^8^ (Table 1).

**Table 1:** Histopathological grading scores.

Scores	Description of the findings
0	Histological findings are normal
1	Loss of crypt architecture and villus shortening, sparse inflammatory cell infiltration, presence of vacuolization and edema in the mucous layer normal muscle layer
2	Villus blunting with flattened and vacuolated cells, crypt necrosis, moderate inflammatory cell infiltration, vacuolization and edema in the mucosal and submucosal layers and normal muscle layer
3	Villus blunting with flattened and vacuolated cells, crypt necrosis, intense inflammatory cell infiltration, vacuolization, and edema in the mucosal and submucosal layers and muscle layer showing edema, vacuolization, and neutrophilic infiltration

Source: Elaborated by the authors.

### Immunohistochemical analysis

Sections (4-µm thick) were prepared from paraffin-embedded intestinal tissues (duodenum). After deparaffinization, antigens were recovered by incubating the slides in citrate buffer (pH 6) for 20 min at 95°C. Endogenous peroxidase was blocked with 3% H_2_O_2_ for 10 min. Sections were then incubated with Muc-2 (Santa Cruz) for 2 h. Sections were then incubated for 30 min with polymer (K4061, DAKO). Antibody binding sites were visualized by incubating the samples with 3,3’-diaminobenzidine (DAB, DAKO) and hydrogen peroxide (H_2_O_2_) to visualize antibody binding. Sections incubated with an antibody diluent and without a primary antibody were used as negative controls. The amount of DAB reaction product from immunostaining was quantified using digital images obtained from at least ten different areas of each section (from four specimens per group) at 400× magnification, analyzed with Adobe Photoshop software.

The results are expressed as the percentage of immunopositive area, calculated by dividing the DAB-positive staining (immunostaining-positive pixels) by the number of pixels per total tissue image multiplied by 100, as previously described^9^.

### Statistical analysis

Data were analyzed using descriptive (mean and standard deviation) and analytical statistics using parametric tests such as ANOVA, followed by a Bonferroni post-test and non-parametric Kruskal–Wallis’ test at a 5% significance level (Graph Pad Prism 6.01 software).

## Results

### Animal weight

**Figure 1:**
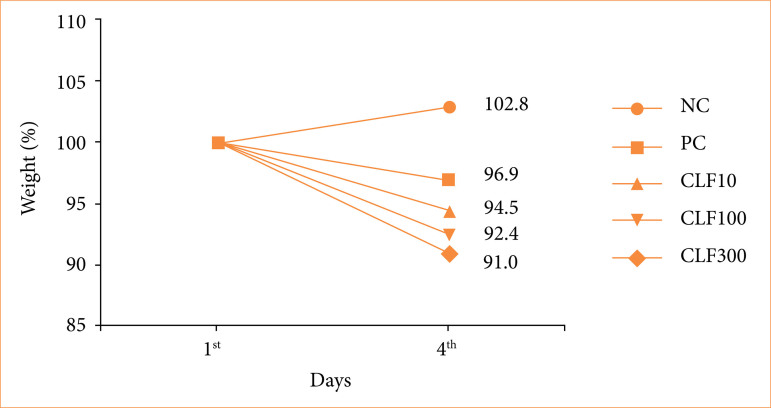
Weight (%). Days: 1 and 4.

NC: negative control; PC: positive control; CLF: *Commiphora leptophloeos* leaves. Source: Elaborated by the authors.

Over the course of three days, animals in the NC group exhibited a weight increase of 2.8% (final weight 102.8% relative to the initial weight, set at 100%). As expected, 5-FU administration resulted in weight loss, with the PC group showing a decrease of 3.1% (final weight 96.9%). Administration of CLF at different doses did not prevent weight loss. In fact, weight loss was dose-dependent, with final weights of 94.5, 92.4, and 91% for the CLF 10, CLF 100, and CLF 300 groups, respectively (Fig. 1).

### Leukocyte count and biochemical data

Leukocyte counts remained within the reference range in all groups treated with the extract at different doses. Similarly, biochemical markers, including AST/TGO, ALT/TGP, and creatinine, were within normal reference parameters for all the three doses (Table 2).

**Table 2:** Global white blood cell count and biochemical: AST/TGO, ALT/TGP and creatinine.

Groups	Global white blood cell count (10^3^/uL)	AST/TGO (U/L)	ALT/TGP (U/L)	Creatinine (mg/dL)
CLF 10	2.68 + 1.47	63.5 + 35.64	32.66 + 4.04	0.12 + 0.04
CLF 100	3.628 + 1.31	127.75 + 55.96	36.5 + 2.12	0.31 + 0.19
CLF 300	1.914 + 0.30	214 + 117.28	3.5 + 14.84	0.76 + 0.39
Reference data (minimum and maximum)	1.785–10.821	51–261	20–150	0.2–1.2

AST: aspartate aminotransferase; TGO: transaminase glutamic-oxaloacetic; ALT: alanine aminotransferase; TGP: transaminase glutamic-pyruvic. Source: Elaborated by the authors.

### Histopathological analysis

**Figure 2:**
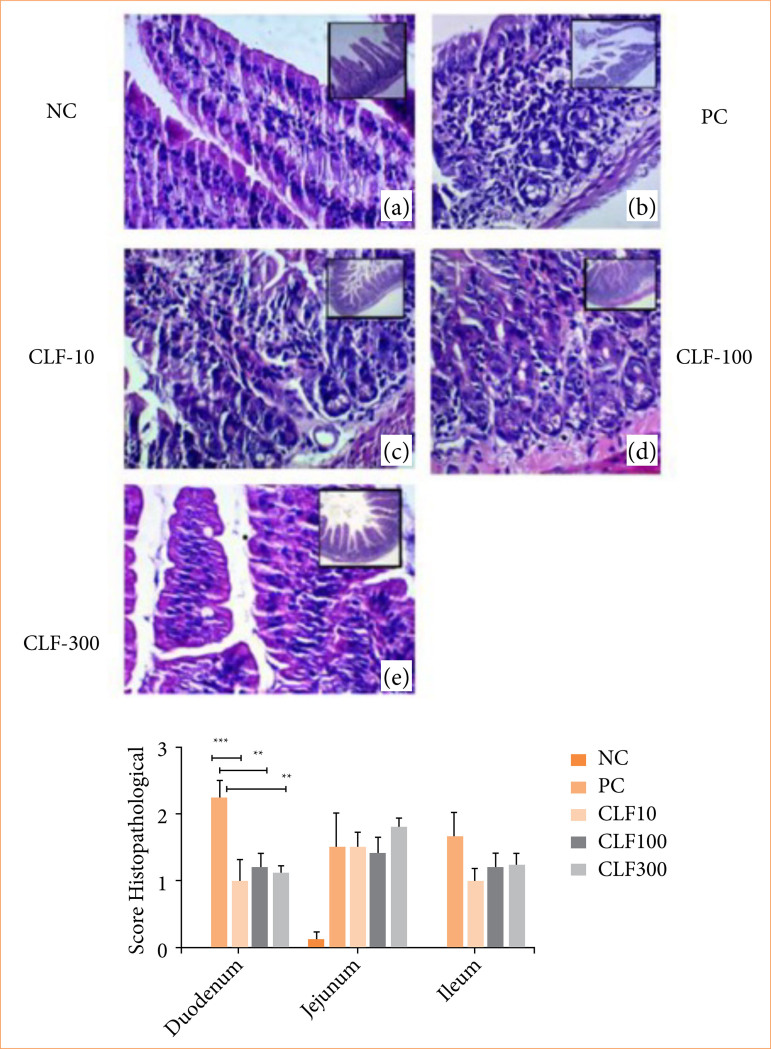
Histopathological analysis of the duodenum from animals in control and treated groups. (a) The NC group shows preserved architecture of villi, crypts, and overall tissue structure. (b) The CP group displays significant loss of duodenal architecture, including crypt destruction, villous shortening, and necrosis. In treated groups, (c) CLF 10 exhibits partial preservation of villous structure, although some areas show morphological damage. (d) In CLF 100, there are marked architectural disorganization and structural alteration of the villi. (e) In CLF 300, villous shortening and deformation are observed. Histopathological analysis: duodenum, jejunum and ileum.

NC: negative control; PC: positive control; CLF: *Commiphora leptophloeos* leaves; **p < 0.01; ***p < 0.001. Source: Elaborated by the authors.

Histopathological evaluation of the small intestine segments revealed that the PC group presented significantly higher lesion scores in the duodenum, with a median score of 2 (2–2.75), compared to the NC group, which had a median score of 0 (0–0) (*p* < 0.001). The CLF 10 group also showed reduced scores 1 (1–1.5), although with statistically significant difference compared to the PC group (*p* < 0.001). Treatment with the CLF significantly reduced duodenal scores at doses of 100 mg/kg score 1 (1–1.5), CLF 300 mg/kg score 1 (1 –1.25) (*p* < 0.01 *versus* PC) (Fig. 2).

In the jejunum, scores were also comparable among groups, without statistically significant differences. The PC group had a median of 1 (1–1.5), while NC score 0 (0–0.75), CLF 10 mg/kg score 1.5 (1–2), CLF 100 mg/kg score 1 (1–2), and CLF 300 mg/kg score 2 (1.5–2).

#### Histopathological analysis of the jejunum from animals in control and treated groups

**Figure 3:**
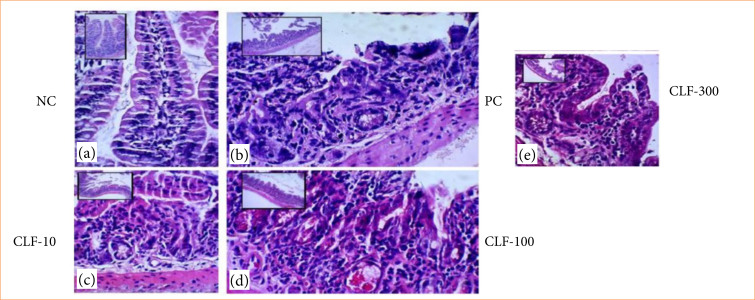
Histopathological analysis of the jejunum from animals in control and treated groups. (a) The CN group displays preserved structural integrity of the tissue, including crypts, villi, and muscle layer. (b) In the CP group, there is clear evidence of crypt architectural loss, villous shortening, and necrosis. In the treated groups, (c) CLF 10 shows areas of villous microvilli deformation, crypt structural damage, and necrotic zones. (d) CLF 100 presents better tissue preservation, but villous shortening is still observed. (e) In CLF 300, there are intense villous shortening and crypt loss with evident necrotic areas.

NC: negative control; PC: positive control; CLF: *Commiphora leptophloeos* leaves. Source: Elaborated by the authors.

The NC group displays preserved structural integrity of the tissue, including crypts, villi, and muscle layer. In the CP group, there was clear evidence of crypt architectural loss, villous shortening, and necrosis. In the treated groups, CLF 10 showed areas of villous microvilli deformation, crypt structural damage, and necrotic zones. CLF-100 presented better tissue preservation, but villous shortening was still observed. In CLF 300, there were intense villous shortening and crypt loss with evident necrotic areas (Fig. 3).

In the ileum, scores were also comparable among groups, without statistically significant differences. The PC group had a median of 2 (1.5–2), while NC 0 (0–0), CLF 10 mg/kg score 1 (1–1.25), 100 mg/kg score 1 (1–1.5) and 300 mg/kg score 1 (1–1.5).

#### Histopathological analysis of the ileum from animals in control and treated groups

**Figure 4:**
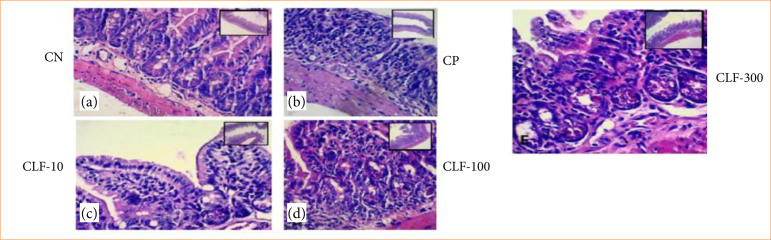
Histopathological analysis of the ileum from animals in control and treated groups. (a) The CN group shows preserved architecture of villi, crypts, and tissue structure. (b) The CP group exhibits significant loss of intestinal architecture, with crypt destruction, villous shortening, and necrosis. In treated groups, (c) CLF 10 shows shortening of villi and inflammatory cell infiltration. In (d) CLF 100, villous shortening, crypt loss, and moderate inflammatory infiltration are observed. (e) CLF 300 presents the most severe damage, with marked loss of villous and crypt architecture.

NC: negative control; PC: positive control; CLF: *Commiphora leptophloeos* leaves. Source: Elaborated by the authors.

The CN group showed preserved architecture of villi, crypts, and tissue structure. The CP group exhibited significant loss of intestinal architecture, with crypt destruction, villous shortening, and necrosis. In treated groups, CLF 10 showed shortening of villi and inflammatory cell infiltration. In CLF 100, villous shortening, crypt loss, and moderate inflammatory infiltration were observed. CLF 300 presented the most severe damage, with marked loss of villous and crypt architecture (Fig. 4).

### Imunohistochemistry: MUC-2

**Figure 5:**
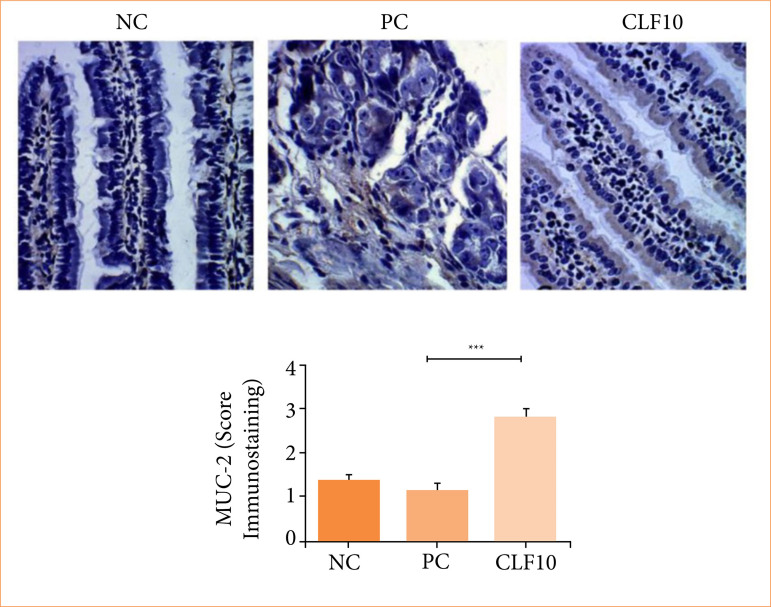
Immunohistochemical analysis to evaluate MUC-2. Duodenum.

NC: negative control; PC: positive control; CLF: *Commiphora leptophloeos* leaves; **p < 0.01; ***p < 0.001. Source: Elaborated by the authors.

The immunostaining findings for MUC-2 in the duodenum showed a score of 1.6 (1.2–1.6) in the NC group. In the CLF 10 group, the score was 3 (3–3), *p* < 0.001, when compared to the PC group, which had a score of 1.2 (0.8–1.6). This indicates that the CLF 10 extract was able to increase mucin immunostaining (Fig. 5).

## Discussion

Previous data on the characterization of the hydroethanolic extract of CLF by mass spectrometry identified the presence of compounds such as phenolic acids, glycosylated flavonoids derived from quercetin, luteolin, and apigenin, and condensed tannins derived from catechin. Furthermore, two flavonoids were isolated by countercurrent chromatography, reinforcing the phenolic profile of the extract^10,11^. The anti-inflammatory action of the extract can be attributed to the presence of these compounds.

The findings of this study demonstrated that the *C. leptophloeos* extract, although did not impact the weight loss caused by 5-FU, showed slight protective activity, especially at lower doses in the treated groups. This is evidenced primarily by histopathological and immunohistochemical analyses, which showed lower degrees of injury in the duodenum, jejunum, and ileum compared to the positive control group. Histopathological analysis revealed partial preservation of the intestinal architecture and absence of damage to the muscular layer. Immunohistochemical analysis, however, revealed an increase in MUC-2 production after treatment with 10 mg/kg (CLF 10), suggesting a possible protective effect of the extract and stimulation of the intestinal mucosal barrier.

These observed effects are consistent with the molecular mechanisms already described for flavonoids present in the extract, such as apigenin, quercetin, and luteolin, which are known to modulate inflammation and promote intestinal mucosal integrity. Therefore, the findings of this study reinforce the existing literature on the anti-inflammatory potential of these natural compounds.

Flavonoids are known for their anti-inflammatory properties. The presence of a planar ring with unsaturation between carbons C2 and C3, as well as hydroxyl groups at the 3’ and 4’ positions of ring B, is crucial to confer this activity. Thus, several flavonoids are capable of reducing the production of prostaglandins, leukotrienes, and nitric oxide, inhibiting potent inflammatory markers such as arachidonic acid, phospholipase A2, cyclooxygenase, and nitric oxide.

Among flavonoids, apigenin has been shown to decrease TNF-α-induced mRNA levels and, therefore, reduce the expression of cell adhesion molecules such as ICAM-1, E-selectin, and VCAM-1 in endothelial cells. Furthermore, inhibition of TNF-α-induced IL-1β, IL-6, and prostaglandin E2 was observed in apigenin-pretreated cells12. Studies demonstrate that quercetin has diverse pharmacological activities, including anti-SARS-CoV-2, antioxidant, anticancer, antiaging, antiviral, and anti-inflammatory properties^13^. Quercetin’s anti-inflammatory activity can be attributed to the inhibition of neutrophil infiltration, as well as the apoptosis of active neutrophils and the reduction of serum levels of pro-inflammatory cytokines^14^. Quercetin is also able to block heat shock factor, reducing the expression of the HSP70 protein and, consequently, heat-induced damage^12^. Phenolic acids are strong antioxidants due to the presence of hydroxyl groups in their structure, which contribute to their anticancer, anti-inflammatory, and cardioprotective potential. The anti-inflammatory activity was attributed to the reduction of pro-inflammatory cytokines^15^.

Some flavonoids present in the extract, such as apigenin, luteolin, and quercetin, may exhibit cytotoxicity at high concentrations, especially in normal human cells such as lung embryonic fibroblasts and human umbilical vein endothelial cells. Cytotoxicity occurred in a dose-dependent manner. Among the compounds evaluated, luteolin, 3-hydroxyflavone, and apigenin were the most cytotoxic. Cytotoxicity was attributed to the increase in intracellular reactive oxygen species, indicating that the pro-oxidant effect of these flavonoids may outweigh their antioxidant properties when administered at high doses^16^.

## Conclusion

The oral administration of a hydroethanolic extract of CLF 10 mg/kg protected the duodenal segment of Swiss mice from 5-FU-induced intestinal mucositis, likely by increasing mucin production.

## Data availability statement

The data are available at https://repositorio.ufrn.br/handle/123456789/66185
